# Mechanisms of *Staphylococcus aureus* survival of trimethoprim-sulfamethoxazole-induced thymineless death

**DOI:** 10.1128/mbio.01634-24

**Published:** 2024-10-24

**Authors:** Lauren J. Gonsalves, Allyson Tran, Tessa Gardiner, Tiia Freeman, Angshita Dutta, Carson J. Miller, Sharon McNamara, Adam Waalkes, Dustin R. Long, Stephen J. Salipante, Lucas R. Hoffman, Daniel J. Wolter

**Affiliations:** 1 Department of Microbiology, University of Washington, Seattle, Washington, USA; 2 Division of Pulmonary and Sleep Medicine, Department of Pediatrics, University of Washington, Seattle, Washington, USA; 3 Pulmonary Division, Seattle Children’s Hospital, Seattle, Washington, USA; 4 Department of Laboratory Medicine and Pathology, University of Washington, Seattle, Washington, USA; 5 Division of Critical Care Medicine, Department of Anesthesiology and Pain Medicine, University of Washington, Seattle, Washington, USA; Emory University School of Medicine, Atlanta, Georgia, USA

**Keywords:** *Staphylococcus aureus*, thymineless death, antibiotic resistance, antifolate drugs, persistence

## Abstract

Trimethoprim-sulfamethoxazole (SXT) is commonly used to treat diverse *Staphylococcus aureus* infections, including those associated with cystic fibrosis (CF) pulmonary disease. Studies with *Escherichia coli* found that SXT impairs tetrahydrofolate production, leading to DNA damage, stress response induction, and accumulation of reactive oxygen species (ROS) in a process known as thymineless death (TLD). TLD survival can occur through the uptake of exogenous thymidine, countering the effects of SXT; however, a growing body of research has implicated central metabolism as another potentially important determinant of bacterial survival of SXT and other antibiotics. Here, we conducted studies to better understand the mechanisms of TLD survival in *S. aureus*. We found that thymidine abundances in CF sputum were insufficient to prevent TLD of *S. aureus*, highlighting the importance of alternative survival mechanisms *in vivo*. In *S. aureus* cultured *in vitro* with SXT and low thymidine, we frequently identified adaptive mutations in genes encoding carbohydrate, nucleotide, and amino acid metabolism, supporting reduced metabolism as a common survival mechanism. Although intracellular ROS levels rose with SXT treatment *in vitro*, survival was not improved in the presence of ROS scavengers, unlike in *E. coli*. SXT challenge induced the SOS response, which was alleviated by added thymidine. Finally, an inactivating mutation in the phosphotransferase gene *ptsI* conferred both limitation in cellular ATP and improved survival against TLD. Collectively, these results suggest that alterations in core metabolic functions, particularly those that reduce ATP levels, predominantly confer *S. aureus* survival and persistence during SXT treatment, potentially identifying novel targets for co-treatment.

**IMPORTANCE:**

*Staphylococcus aureus* is a ubiquitous organism and one of the leading causes of human infections, many of which are difficult to treat due to persistence, antibiotic resistance, or antibiotic tolerance. As our arsenal of effective antibiotics dwindles, the need for improved treatments becomes increasingly urgent, necessitating a better understanding of the precise mechanisms by which pathogens evade our most critical antimicrobial agents. Here, we report a systematic characterization of the mechanisms of *S. aureus* survival to treatment with the first-line antistaphylococcal antibiotic trimethoprim-sulfamethoxazole, identifying pathways and candidate targets for enhancing the efficacy of available antimicrobial agents.

## INTRODUCTION

The opportunistic pathogen *Staphylococcus aureus* causes diverse human and animal infections, including acute infections, such as those of skin and soft tissues, as well as chronic infections such as those of bone and the respiratory tract (1–3). Among the best-studied chronic *S. aureus* infections are those that occur in the airways of people with cystic fibrosis (PwCF), for whom lung disease is the primary cause of morbidity and mortality (4, 5). *S. aureus* is one of the earliest and most common pathogens isolated from cystic fibrosis (CF) secretions (6–9), and the antistaphylococcal agent trimethoprim-sulfamethoxazole (SXT) is a first-line, orally available therapy for these infections, especially for methicillin-resistant *S. aureus* (MRSA) (10, 11).

SXT synergistically interrupts two separate steps in folate biosynthesis (12), which is required to produce diverse metabolites. Among these, impaired synthesis of deoxythymidine monophosphate (dTMP), a precursor to the triphosphate thymidine nucleotide, is lethal (13–16). Thymidine starvation results in defective DNA synthesis and repair in a process known as thymineless death (TLD) first identified over 60 years ago (17, 18). Most research on TLD and thymidine starvation was conducted in *Escherichia coli* (17–20), suggesting a model in which thymidine depletion increased the production of reactive oxygen species (ROS), resulting in lethal DNA damage (21, 22). However, the involvement of ROS in lethality by this and other antibiotics remains controversial (23, 24) and may vary between bacterial species (25). Clarifying the mechanisms of *S. aureus* TLD survival is a key step toward a broader mechanistic understanding of antibiotic-mediated killing.

Relatively few SXT-specific survival mechanisms have been described in *S. aureus*. Target site mutations in dihydrofolate reductase and dihydropteroate synthase cause trimethoprim and sulfonamide resistance, respectively (26). Some bacteria, including *S. aureus*, can survive both agents by taking up exogenous thymidine (27–29), thereby reducing or eliminating the bactericidal activity of SXT. SXT resistance is also observed in thymidine-dependent small colony variants (TD-SCVs), which are commonly detected in PwCF after SXT treatment (30, 31), and carry mutations in the thymidylate synthase gene (*thyA*) encoding a critical enzyme for dTMP production (32, 33). TD-SCVs survive TLD by upregulating a thymidine transporter (33).

The persistence of *S. aureus* in PwCF despite SXT treatment and the common detection of TD-SCVs in such individuals suggest that free thymidine concentrations in CF respiratory secretions should be sufficient to prevent TLD among both TD-SCVs and at least some wild-type *S. aureus* cells during SXT treatment (30, 31). However, alternative mechanisms of TLD survival beyond exogenous thymidine uptake may contribute to persistence. For example, SCVs carrying mutations in genes related to electron transport, such as menadione and hemin synthesis, are also observed among CF patients treated with SXT (34–36). Whether SXT can also select for these metabolic mutants is unknown, but a growing body of evidence has linked attenuated metabolic rates to survival of diverse environmental stresses, including antibiotics, immunity, and intracellular defenses (32, 37–39). These observations suggest that *S. aureus* may possess multiple, undefined mechanisms for surviving TLD that include core metabolic alterations.

Here, we explored the relationship between thymidine availability and *S. aureus* survival strategies during exposure to SXT to thoroughly define the mechanisms responsible for TLD. We hypothesized that different cellular processes would be involved in surviving TLD depending on exogenous thymidine levels. We show that *S. aureus* utilizes numerous strategies to survive SXT and the resulting TLD, beyond mutations in folate biosynthetic target sites and *thyA*, particularly under low thymidine conditions. We also find that ROS production is not the primary driver of cell death in *S. aureus*, in contrast with prior studies conducted in *E. coli*.

## RESULTS

### Thymidine metabolism and uptake play important roles in SXT killing

TD-SCVs escape TLD through the uptake of exogenous thymidine and its analogs (33, 40). Because SXT treatment phenocopies TD-SCV-causative mutations by impairing dTMP synthesis, *S. aureus* may also survive SXT treatment in clinical infections (such as those in CF airways) by increasing the uptake of available thymidine. However, neither the thymidine concentrations required for *S. aureus* to survive SXT nor those present in CF sputum have been defined.

**Fig 1:**
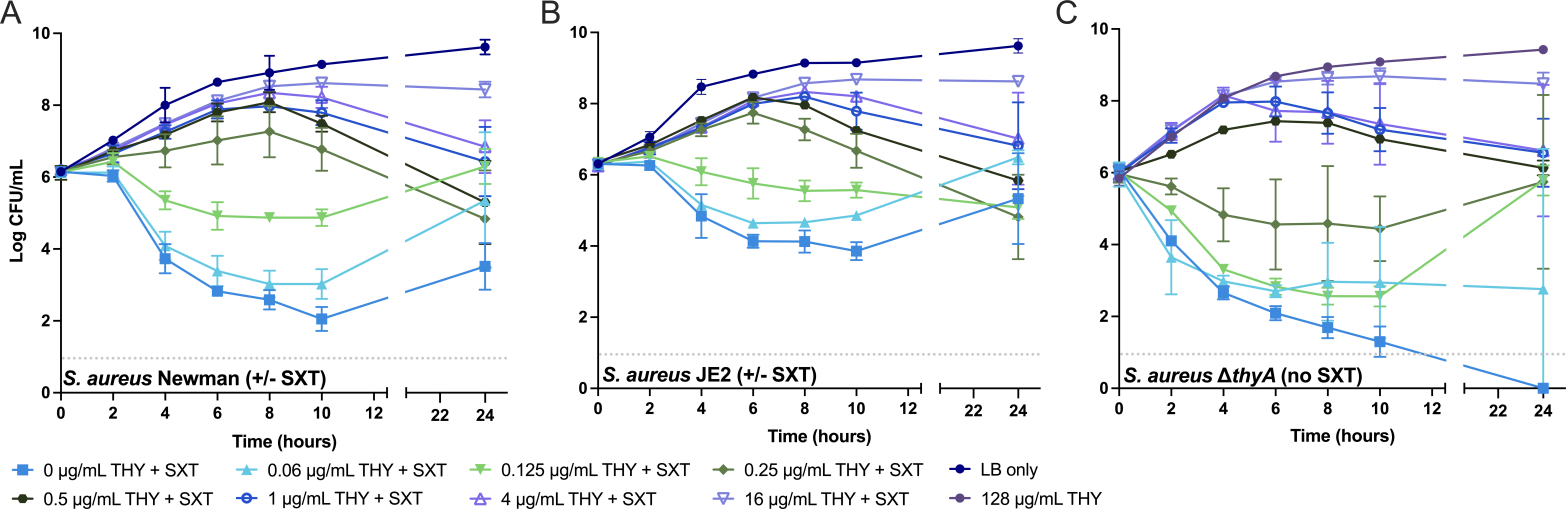
Kinetics of survival of two laboratory *S. aureus* strains and a TD-SCV mutant in the presence and absence both of trimethoprim-sulfamethoxazole and thymidine supplementation. *S. aureus* strains (**A**) Newman, (**B**) JE2, and (**C**) Newman-derived ∆*thyA* cultured in LB over 24 h. Each strain was supplemented with the concentrations of thymidine (THY) indicated in the legend (0–16 µg/mL); strains Newman and JE2 were treated with 8 µg/mL TMP and 152 µg/mL SMX; ∆*thyA* was not treated with SXT (indicated in panel C) due to its natural ability to undergo TLD in the absence of thymidine. A control experiment including supplementation with 128 µg/mL THY to simulate wild-type-like growth was included for ∆*thyA* only (**C**). Data represent mean values ± SD (*n* ≥ 2).

We, therefore, determined the relationship between thymidine concentration and *S. aureus* survival during *in vitro* culture with and without SXT. Methicillin-susceptible *S. aureus* strain Newman, methicillin-resistant strain JE2, and Newman ∆*thyA* (TD-SCV) were cultured over 24 h at varying thymidine concentrations (0–16 µg/mL), with and without clinically relevant SXT concentrations (8- to 16-fold above the parental MICs) (41). We found the thymidine concentration required to survive SXT *in vitro* differed little among the two non-SCVs (Fig. 1A and B); concentrations ≥ 0.25 µg/mL supported increasing cell densities over 24 h with a decline after 8–10 h, while cell densities uniformly declined, but remained above the limit of detection, at thymidine concentrations below 0.25 µg/mL. After a period of rapid cell death following SXT treatment in the absence of exogenous thymidine, culturable cell counts quickly increased with the addition of 1 µg/mL of thymidine at 4 or 6 h (Fig. S1) time points, indicating either that damaged cells may be rapidly repaired and replicate or that a static and uninjured subset of the cell population responds to exogenous thymidine. Despite the two strains having similar MICs to SXT (Newman MIC = 0.5 µg/mL, JE2 MIC = 0.25 µg/mL), Newman was more susceptible to SXT killing than JE2 (~4 log CFU/mL decrease for Newman versus ~2 log CFU/mL decrease for JE2 at 10 h under low thymidine conditions), underscoring a functional distinction between cell death and MICs. Survival patterns of the ∆*thyA* mutant (Fig. 1C) differed slightly from those of non-SCV strains only under low-thymidine conditions. Compared with wild-type parent Newman, which might produce low levels of thymidine due to incomplete folate inhibition with SXT, ∆*thyA* was entirely dependent on exogenous thymidine for viability, ultimately declining to densities below the limit of detection with 0 µg/mL thymidine, but with improved survival at 0.25 µg/mL. These results demonstrated that, under these experimental conditions, 0.25 µg/mL of thymidine was sufficient for all tested *S. aureus* strains to survive SXT challenge or, in the case of ∆*thyA*, TLD.

**Fig 2:**
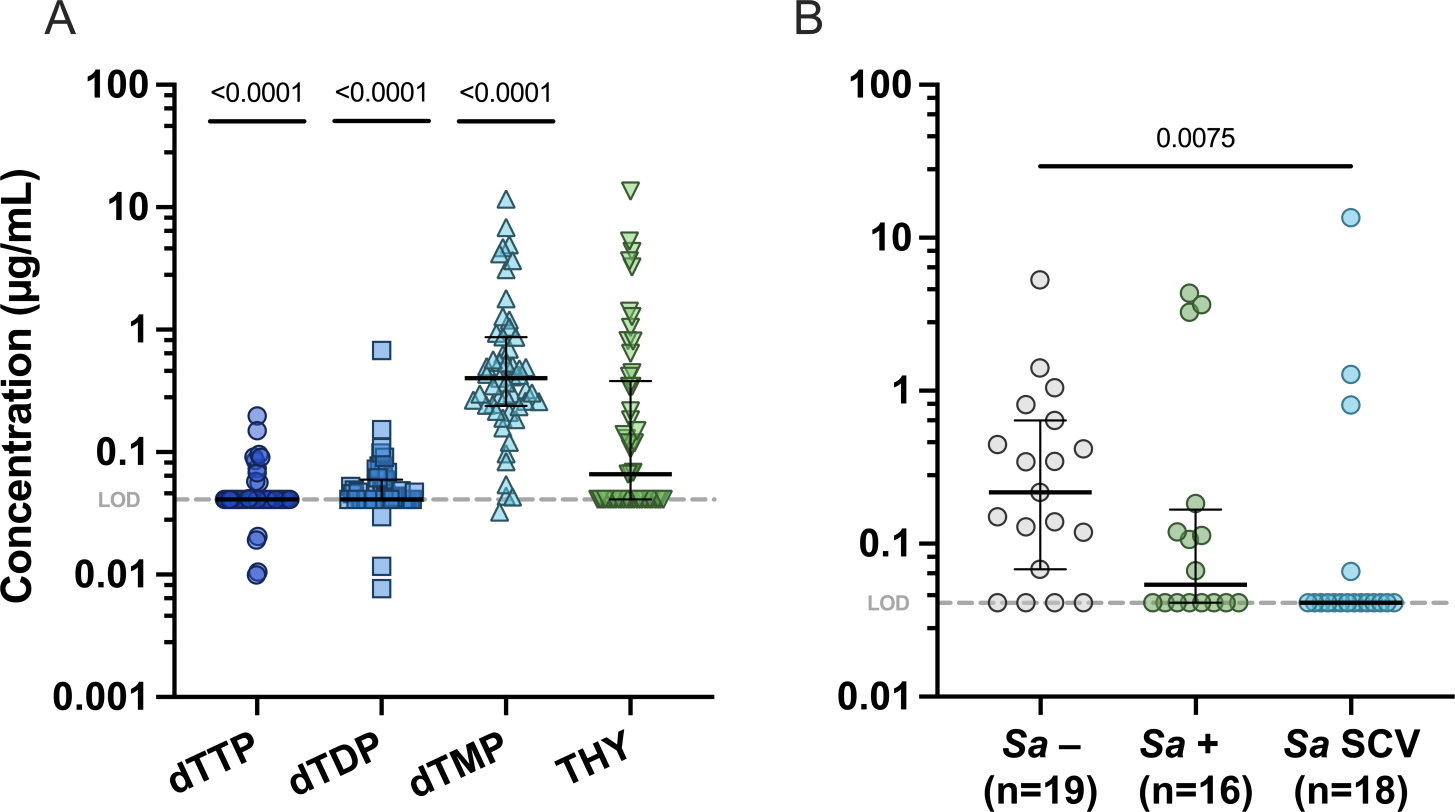
Concentrations of free thymidine and its analogs in sputum samples from PwCF. (**A**) LC-MS/MS analysis of free deoxythymidine triphosphate, deoxythymidine diphosphate, deoxythymidine monophosphate, and thymidine in 53 CF sputum samples. Significance was determined using Kruskal-Wallis and Dunn’s multiple comparison tests; significant *P*-values are reported. (**B**) All analyzed samples were categorized as *S. aureus*-negative, *S. aureus*-positive (non-SCV), or *S. aureus* SCV-positive and the corresponding thymidine concentration as determined by LC-MS/MS; all data are reported as median with interquartile range. For panels A and B, the dotted line indicates the 0.041 µg/mL limit of detection. Significance was determined using either the (**A**) Wilcoxon signed rank test (theoretical mean of 0.25 µg/mL) or the (**B**) Kruskal-Wallis test for multiple comparisons (right); only significant *P*-values are reported.

We reasoned that, if *S. aureus* primarily survives SXT through thymidine uptake during CF respiratory infection, thymidine concentrations in infected secretions should be ≥0.25 µg/mL. However, the concentrations of that nucleoside and its analogs in human tissues are unknown (27, 39, 40). Therefore, to assess the relationship between *S. aureus* detection and thymidine concentration in CF, we analyzed 53 CF sputum samples using mass spectrometry to quantify the nucleoside thymidine and the monophosphate, diphosphate (dTDP), and triphosphate (dTTP) nucleotides and cultured for *S. aureus*. dTTP and dTDP were generally undetectable, while measurements ranged from below the limit of detection (0.041 µg/mL) to either 13.55 µg/mL for thymidine or 11.69 µg/mL for dTMP (Fig. 2A). Although dTMP was detected in many sputum samples, this nucleotide cannot support *thyA* mutant growth or antagonize the activity of SXT on its own (Fig. S3). While thymidine levels in all samples (median = 0.066) were not statistically different than 0.25 µg/mL (*P* = 0.173), only 28% of samples (15/53) contained thymidine levels ≥ 0.25 µg/mL, with an even smaller proportion (6/34, ~18%) of *S. aureus* culture-positive samples reaching this concentration. Median sputum thymidine concentration was not significantly lower in *S. aureus* culture-positive samples (median = 0.054) compared with culture-negative samples (median = 0.217); median sputum concentration was significantly lower in *S. aureus* SCV-positive samples (median = 0.041), which included TD-SCVs, relative to *S. aureus*-negative samples (*P* = 0.0075) (Fig. 2B), possibly as a result of SCVs consuming the exogenous supply to grow and survive. These data suggest that while present at detectable levels in some samples, CF sputum thymidine abundances alone may frequently be insufficient to confer *S. aureus* survival of SXT.

**Fig 3:**
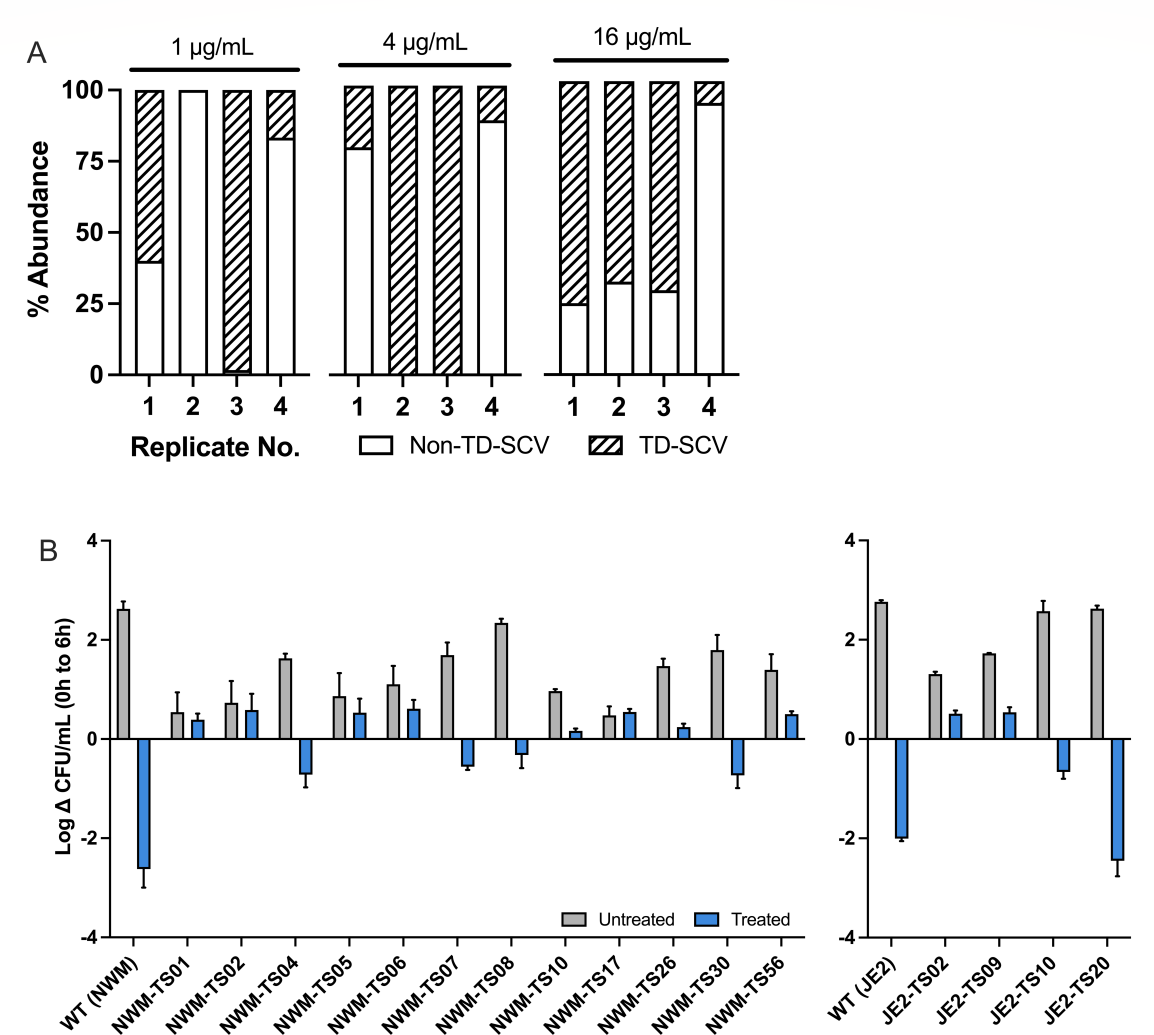
Characteristics of adaptive mutants cultured from LB following SXT challenge. (**A**) Proportion of colonies exhibiting the TD-SCV morphology following 120 h of SXT exposure of Newman under high thymidine conditions (1, 4, and 16 µg/mL). Phenotypes were determined by plating on blood agar, and total bacterial abundances were measured on chocolate agar plates (*n* = 4). (**B**) Survival of a subset of adaptive mutants isolated after 24 h from low thymidine concentrations relative to parental counterpart with re-exposure to SXT. Shown is change in log CFU/mL between 0 and 6 h in LB with the addition of SXT and in the absence of supplemental thymidine. Data represent mean ± SD (*n* ≥ 2). The isolates listed above contain nonsynonymous mutations in the following genes (Table S1): *memE* (NWM-TS01), *menF* (NWM-TS02), *hemE* (NWM-TS04), *aroB* (NWM-TS05), *aroA2* (NWM-TS06), *hemH* (NWM-TS07), *ptsI* (NWM-TS08), *thiN* (NWM-TS10), *ndk* and *hepT* (NWM-TS17), *tkt* (NWM-TS56), *pyk* (JE2-TS02), *pta/eutD* (JE2-TS09), *nrdE* (JE2-TS10), and *deoB* (JE2-TS20); no mutations were detected in isolates NWM-TS26 and NWM-TS30.

### Mutations selected by the SXT challenge vary by thymidine availability

To determine how thymidine availability influences *S. aureus* mutant selection by SXT, strains Newman and JE2 were again cultured in the presence and absence of SXT at clinically relevant concentrations and 8- to 16-fold above the parental MICs (super-inhibitory) and with various thymidine concentrations. At 24 and 120 h, we isolated surviving variants for whole-genome sequencing (WGS). We observed that colony phenotypes from these cultures varied more after selection under low thymidine compared to those from high thymidine, despite originating from a phenotypically homogeneous inoculum (Fig. S2A and C).

After selection with higher thymidine concentrations, colonies generally exhibited only normal (Fig. S2B and D, right) or classical TD-SCV morphologies (transparent or fried egg-like colonies) on blood agar. With Newman, WGS identified *thyA* mutant isolates in 11 of 12 replicate cultures, predominating in cell populations at 120 h in most replicates (Fig. 3A). Interestingly, *thyA* mutants were never detected among four separate JE2 cultures when grown with any thymidine concentration (data not shown). WGS of mutants selected by SXT with high thymidine largely supported these phenotypic observations, revealing mutations in *deoB* (phosphopentomutase, both Newman and JE2), *dfrA* (dihydrofolate reductase, Newman only), and *thyA* (thymidylate synthase, Newman only), encoding genes involved in folate metabolism (*dfrA*) (13) and thymidine uptake and metabolism (*deoB* and *thyA*) (19, 42).

By contrast, with low thymidine concentrations, cultures yielded a mixture of hyperpigmented colonies (indicative of staphyloxanthin hyperproduction; Fig. S2E), pinpoint-to-small colonies (many surprisingly hemin- or menadione-dependent SCVs; Fig. S1B and D), and large colonies. This phenotypic variation was reflected genetically by WGS analysis of both Newman and JE2 isolates collected at 24 and 120 h (summarized in Table 1). Mutations were observed in diverse genes involved in nucleotide metabolism (*nrdE/F*), electron transport component synthesis (*hepT*, *hemH/E/Y, memE*, and *menF*), translation (*rpoC*), and central energy metabolism (*ptsI*, *thiN, ackA*, *pta*, *aroA/B*, *pdhB*, *pyk*, and *pdhD/IpdA*), in addition to others encoding hypothetical proteins. Genetic alterations in these strains consisted of missense mutations and gene inactivating changes, including nonsense mutations and nucleotide insertions/deletions causing frameshifts (Table S1A and B).

**TABLE 1:** Summary of genes with non-synonymous mutations observed through sequencing of isolates and/or whole cultures after SXT selection with either high or low THY concentrations

Gene name	Pathway	Protein	Time^*a*^	[THY]^*b*^	Strain	Isolate/whole culture
*ptsI*	Central metabolism	Phosphoenolpyruvate phosphotransferase	Early, late	Low	Newman, JE2	Isolate and whole culture
*thiN*		Thiamine pyrophosphokinase	Early, late	Low	Newman	Isolate
*pdhB*		Pyruvate dehydrogenase, beta subunit	Early	Low	JE2	Isolate
*ackA*		Acetate kinase	Early, late	Low	Newman, JE2	Isolate and whole culture
*aroA aroB*		3-Phosphoshikimate 1-carboxyvinyltransferase; 3-dehydropuinate synthase	Early	Low, high	Newman, JE2	Isolate and whole culture
*pta*		Phosphate acetyltransferase	Early, late	Low	Newman, JE2	Isolate and whole culture
*pyk*		Pyruvate kinase	Early, late	Low	Newman, JE2	Isolate and whole culture
*pdhD* *IpdA*		Dihydrolipoamide dehydrogenase	Early, late	Low	Newman, JE2	Isolate
*heptT*	Electron transport chain (ETC) component synthesis	Heptaprenyl diphosphate synthase component II	Early	Low	Newman, JE2	Isolate
*hemH hemE hemY*		Ferrochelatase; uroporphyrinogen decarboxylase; protoporphyrinogen oxidase	Early, late	Low, high	Newman	Isolate and whole culture
*nrdE nrdF*	Nucleotide metabolism	Ribonucleotide-diphosphate reductase, alpha and beta subunits	Early, late	Low	Newman, JE2	Isolate and whole culture
*deoB*		Phosphopentomutase	Late	High	Newman, JE2	Isolate and whole culture
*thyA*		Thymidylate synthase	Late	High	Newman	Isolate and whole culture
*rpoC*	Translation	DNA-directed RNA polymerase, subunit β′	Early, late	Low	Newman, JE2	Isolate and whole culture
*dfrA*	Folate metabolism	Dihydrofolate reductase	Late	High	Newman	Isolate and whole culture

^*a*^ Time: early and late refer to 24 and 120 h, respectively.

^*b*^ [THY]: low includes 0–0.25 µg/mL thymidine conditions; high includes 1–16 µg/mL thymidine conditions.

The culture-based methods we used to select resistant isolates could preferentially identify fixed or stable mutations. To limit this culture-associated bias and better determine relative mutation frequencies, we performed whole-genome sequencing directly on the bacterial populations comprising up to four biological replicate cultures each of Newman and JE2 collected at 24 and 120 h of SXT treatment with varying thymidine concentrations (summarized in Table 1; Tables S2 to S9). With high thymidine at 120 h, a few genes in these populations were commonly mutated among both Newman and JE2 following SXT challenges, including *deoB* and *mvaK1/mvk* (mevalonate kinase). High proportions of variants in *thyA* and *dfrA* were detected from Newman but not JE2, confirming culture results (Fig. 3A). Additional mutations were observed in genes encoding hypothetical proteins at 120 h. With low thymidine at 120 h, gene mutations commonly observed in both Newman and JE2 involved central carbon metabolism (*pta*, *ackA*, and *ptsI*), nucleotide synthesis (*nrdE* and *nrdF*), tRNA biosynthesis and transcription (*rpoC*), and transcriptional regulation (*ctsR*; CsoR-like sulfurtransferase repressor). Similar to strain-level WGS, population-based sequencing identified various types of mutations, even in the same gene under different conditions, including missense mutations, nonsense mutations, and insertions/deletions. These data further support a model wherein survival of SXT in the presence of ample thymidine involves modulation of either uptake or metabolism of nucleotides, whereas survival under thymidine depletion entails changes in core metabolism, growth, and stress responses.

### *S. aureus* deploys diverse strategies to survive SXT-mediated TLD

Several of the mutations selected under low-thymidine conditions affected genes involved in generating ATP or metabolites that participate in glycolysis and the TCA cycle, such as pyruvate (*pdhB, pyk,* and *ptsI*) (43–45), acetyl-coA (*ackA, pta*) (46), and thiamine triphosphate (*thiN*) (47), as well as electron transport chain (ETC) components (e.g., *hepT, memE,* and *hemE*) (48, 49). For a subset of selected mutants carrying mutations in these genes, we assessed whether *S. aureus* survival during SXT exposure under thymidine deplete conditions was impacted (Fig. 3B). Most of the SXT-adapted mutants tested exhibited improved survival relative to wild type upon SXT re-exposure: wild-type viable cell densities decreased ~3 log CFU/mL from 0 to 6 h during SXT treatment, while mutant cell densities generally dropped no more than 1–2 log CFU/mL over the same period. Some tested mutants exhibited a slight increase in density at 6 h with SXT, despite the absence of thymidine. SXT tolerance was confirmed in Newman hemin- and menadione-dependent SCVs containing single gene knockouts (Fig. S4A and B, respectively) and a hyperpigmented JE2 transposon insertion mutant (50) (*hepT*, NE1920; Fig. S4C). Supplementation with hemin or menadione re-established the death of the respective mutants during SXT challenge, indicating that electron transport impairment can confer SXT tolerance.

Relative to wild-type Newman, several tested isolates (*memE, hemE, thiN,* and *hepT* mutants) exhibited growth defects without treatment. Slow bacterial growth was previously linked to generally reduced antibiotic efficacy (51, 52) and could explain survival against TLD. However, we found that Newman cultured in dilute media, which was used to artificially slow growth rates to those resembling ETC mutants, still resulted in its killing by SXT, albeit at a slower rate relative to undiluted LB (Fig. S5). In addition, the growth rates of Newman *ptsI* and JE2 *hepT*::Tn mutants were similar to those of their parental strains grown without SXT and persisted with the addition of SXT. These findings suggest that decreasing the rate of growth improves the survival of SXT, perhaps explaining the isolation of some SXT-adapted isolates carrying mutations in metabolic genes, but factors besides altered growth rate can also protect against TLD.

### The effects of functional alterations in *ptsI* indicate the importance of metabolism in surviving TLD

The above genetic analyses indicated that modulation of core metabolic activities could confer the survival of SXT under thymidine limitation. One of the metabolic gene mutations repeatedly selected from both strains under those conditions occurred in *ptsI* (phosphoenolpyruvate phosphotransferase; Table 1; Tables S2 to S9). The encoded enzyme catalyzes the first step in the phosphotransferase system (PTS), which regulates sugar import and carbohydrate metabolism (44). Mutant NWM-TS08 contains a nucleotide change in *ptsI* (T1625A), resulting in a premature stop codon (Leu542*) (Table S1A). Complementation of NWM-TS08 with a wild-type copy of *ptsI* (NWM-TS08 + pCN34*-ptsI*) restored SXT susceptibility of NWM-TS08 to that of the wild type (Fig. S6), indicating a role for the PTS in mediating susceptibility to SXT. Interestingly, we found no evidence connecting the PTS with folate metabolism from prior published work. However, a single recent study reported that mutations in the *E. coli* PTS conferred general antimicrobial tolerance, although this study did not investigate SXT in particular (53). Because *ptsI* mutants exhibited SXT tolerance but not slow growth, we reasoned that *ptsI* inactivation conferred the survival of SXT through as-yet undefined pathways or factors, and we, therefore, included a *ptsI* mutant (NWM-TS08) in our subsequent experiments to assist with investigating these candidate mechanisms.

### ROS are not required for SXT lethality in *S. aureus*

**Fig 4:**
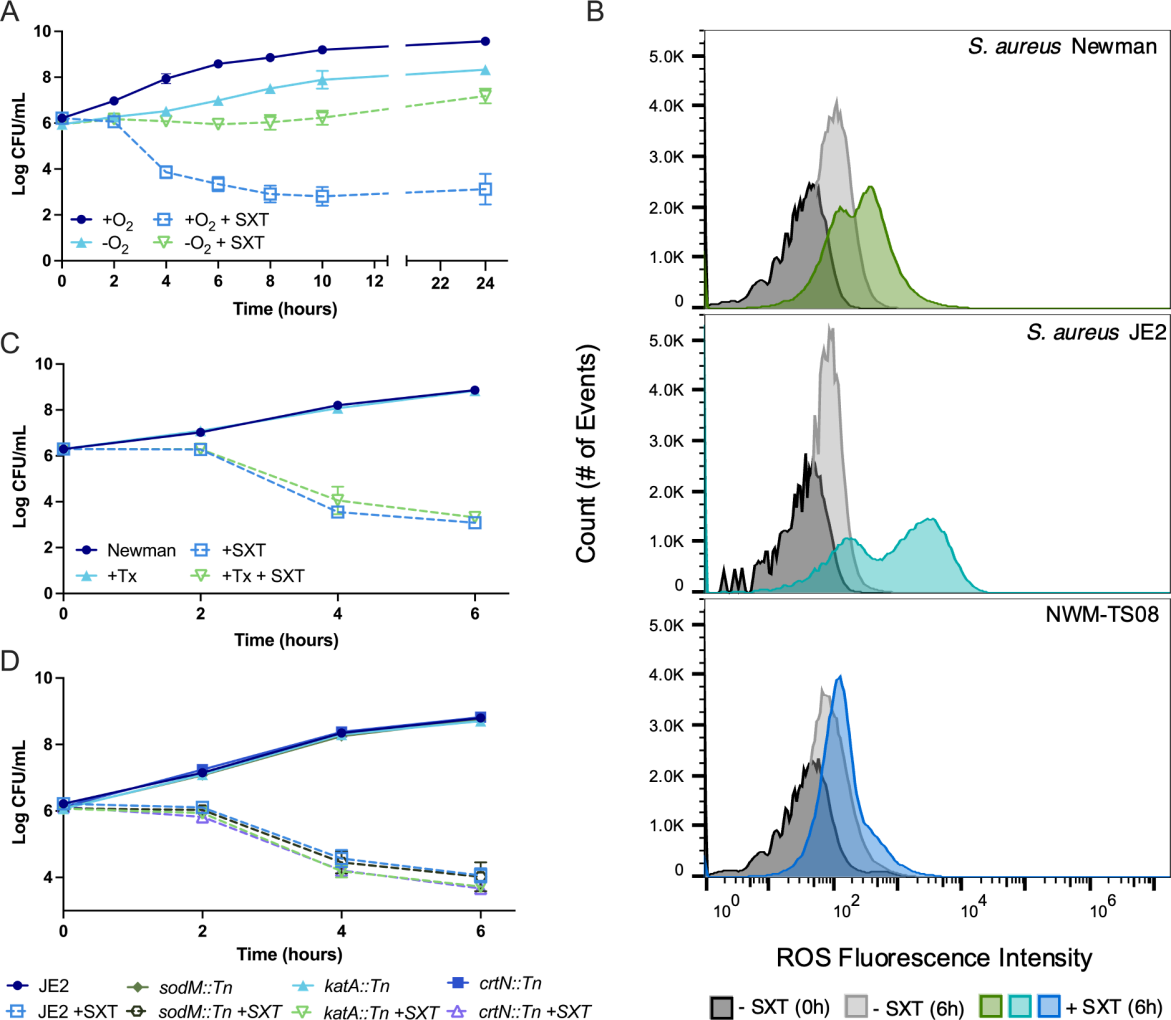
Effect of oxygen and reactive oxygen species on *S. aureus* survival of SXT. (**A**) Survival kinetics of *S. aureus* Newman cultured under aerobic versus anaerobic conditions in LB over 10 h in the absence of additional thymidine and treated with SXT; data are mean ± SD (*n* = 3). (**B**) Intracellular ROS, measured by flow cytometry and fluorescent indicator CMH_2_DCFDA, in *S. aureus* Newman, JE2, and *ptsI* mutant NWM-TS08 with and without SXT at 0 and 6 h. In total, 100,000 events were collected for each condition, where possible. Representative data of at least three replicate experiments. (**C**) Survival kinetics of *S. aureus* Newman with and without ROS scavenger Trolox (Tx) and SXT in LB over 6 h in the absence of added thymidine; SXT-treated conditions are indicated by a dotted line and data are mean ± SD (*n* = 2). (**D**) Survival kinetics of *S. aureus* JE2 carrying transposons in ROS detoxification genes (*sodM*::Tn, *katA*::Tn, and *crtN*::Tn) with and without SXT in LB over 6 h in the absence of additional thymidine; SXT-treated conditions are indicated by a dotted line and data are mean ± SD (*n* = 3).

Recent work, largely using *E. coli,* suggested that ROS play a dominant role in killing by SXT and perhaps other bactericidal antibiotics (21, 22). Because ROS are produced by partial reduction of oxygen at the ETC and are scavenged by staphyloxanthin (54), the selection of ETC-defective and staphyloxanthin-overproducing mutants by SXT challenge suggested that reducing ROS could be another TLD survival strategy. We first tested this hypothesis by determining the requirement for oxygen in SXT antimicrobial activity. We cultured Newman in the absence and presence of oxygen and SXT for up to 24 h (Fig. 4A). We found that anaerobic growth was slower than aerobic growth, but that the addition of SXT without oxygen had minimal effect on cell viability under these conditions, indicating that either oxygen (and potentially ROS) or maximal growth is required for full SXT lethality and TLD in *S. aureus*.

To determine the relationship between ROS and killing by SXT, we then measured intracellular ROS levels during aerobic growth with and without SXT. Using a flow cytometric assay of the broad-range ROS fluorescent indicator CM-H_2_DCFDA [5-(and-6)-chloromethyl-2′,7′-dichlorodihydrofluorescein diacetate], we found that ROS levels in *S. aureus* were higher in the presence of SXT for both JE2 and Newman (Fig. 4B), similar to previously published results (25), compatible with a role for ROS in SXT lethality. Mutant NWM-TS08 exhibited only a subtle increase in intracellular ROS with SXT when compared to Newman and JE2 (Fig. 4B), indicating either that decreased ROS activity supports SXT tolerance or that it is a byproduct of decreased metabolic activity.

We, therefore, tested whether ROS mitigation improved the survival of SXT by treating Newman with the drug in the presence and absence of the membrane-permeable antioxidant Trolox, which scavenges hydroxyl radicals, hydrogen peroxide, and peroxyl radicals. Although Trolox substantially decreased intracellular ROS (Fig. S7), it exerted no substantial effect on either growth or SXT killing in Newman (Fig. 4C). We also measured ROS levels and SXT effects for JE2-derived mutants carrying transposon insertions (50) in genes encoding ROS detoxification enzymes, including catalase (*katA,* NE1366), superoxide dismutase (*sodM*, NE1224), and 4,4′-diapophytoene desaturase (*crtN*, an enzyme in the staphyloxanthin biosynthetic pathway, NE382) and found no effect of impairing ROS detoxification on SXT killing (Fig. 4D). These data indicate that while ROS are increased in the presence of SXT, they are not the primary drivers of SXT-induced cell death in *S. aureus*.

### SXT activates the SOS response in wild-type *S. aureus* but not the *ptsI* mutant

**Fig 5:**
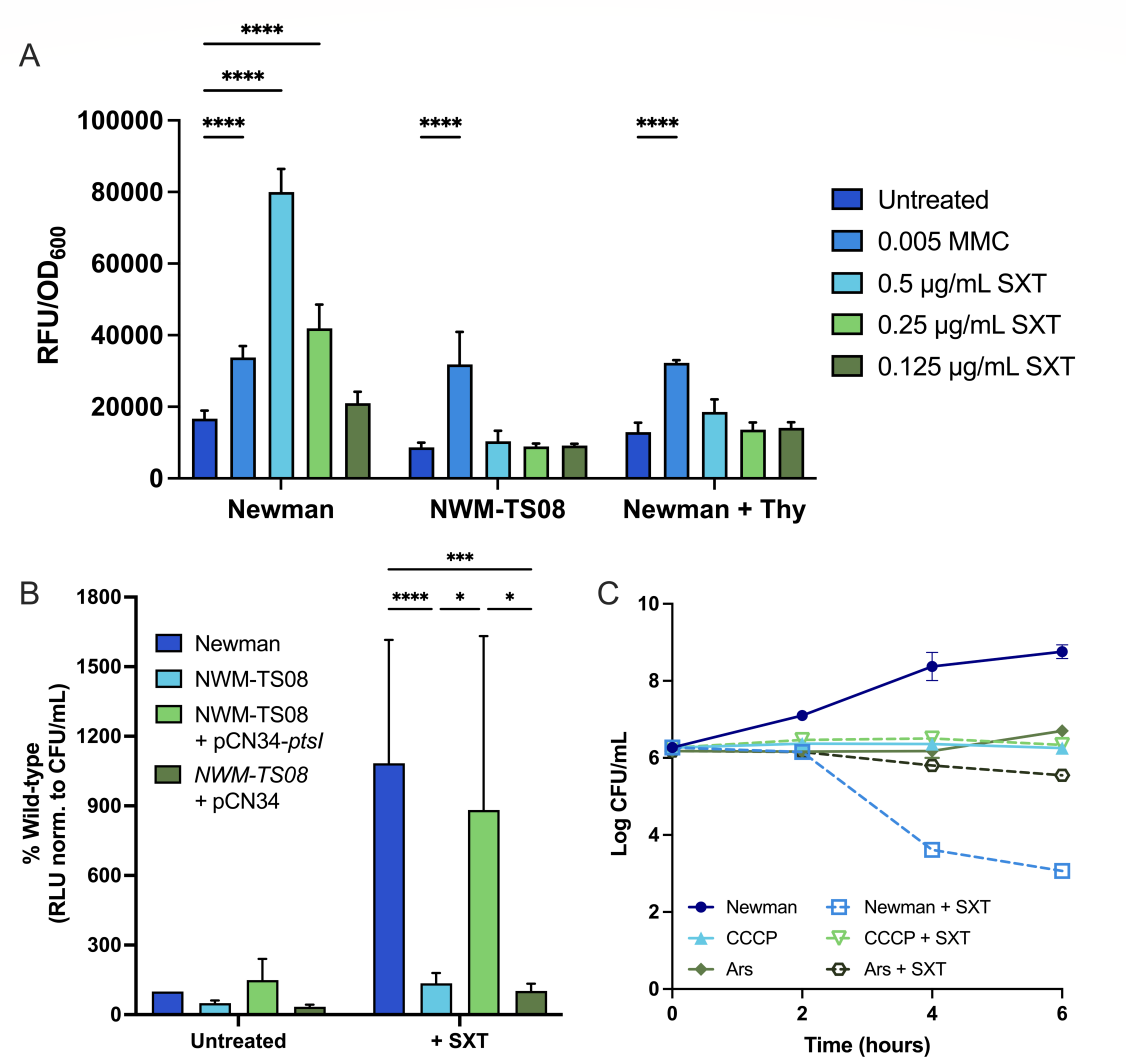
SOS induction, ATP levels, and survival during SXT treatment after ATP depletion. (**A**) *recA*-GFP expression in response to sub-inhibitory levels of SXT (1.25–0.5 µg/mL SXT) or mitomycin C (0.005 µg/mL MMC; control) in Newman (with and without 128 µg/mL thymidine) and NWM-TS08 at 6 h post-SXT exposure; RFU was normalized to OD_600_ and significance was reported as **** when *P* ≤ 0.05 (two-way ANOVA). Data are mean ± SD (*n* ≥ 3). (**B**) Relative whole-culture ATP levels at 2 h post-SXT exposure (RLU normalized to CFU/mL) presented as percentage of wild type for treated and untreated Newman, NWM-TS08, and NWM-TS08 complemented with wild-type *ptsI* (NWM-TS08 + pCN24*-ptsI*); data are mean ± SD (*n* ≥ 3), and significance is reported as *, ***, or **** where *P* ≤ 0.05 (two-way ANOVA). (**C**) Survival kinetics of Newman in LB with and without SXT and added CCCP or arsenate (Ars) as indicated; SXT-treated conditions are indicated by a dotted line and data are mean ± SD (*n* = 3).

DNA damage is considered a sentinel event during TLD in *E. coli* (15, 20, 55) and during SXT-mediated cell death in *S. aureus* (25). In thymidine deplete conditions, DNA strand breaks induce the pleiotropic SOS stress response, generating widespread changes in gene expression and corresponding cellular activities. This response is triggered by RecA binding to damaged DNA, activating RecA, which in turn cleaves SOS repressor LexA, resulting in increased expression of SOS-responsive genes such as those encoding DNA repair enzymes (56, 57). In the absence of sufficient nucleotide pools, attempts to repair DNA promotes further DNA damage and cell death. Because the *ptsI* mutant NWM-TS08 exhibited greatly improved survival against SXT relative to wild type, we compared the SOS response during SXT exposure in these two strains. Using a *recA* promoter-GFP fusion construct, we measured SOS activity at 6 h, during active cell death under our experimental conditions, in Newman and NWM-TS08 with and without sub-inhibitory SXT (Fig. 5A). Experiments with Newman were repeated with excess thymidine (128 µg/mL). As a positive control, strains were also exposed to mitomycin C (0.005 µg/mL), a well-characterized SOS response-inducing agent (58). SOS induction dynamics were indistinguishable among all strains treated with mitomycin C, indicating that all strains and conditions had similar intrinsic SOS response capacities. In contrast, SOS was induced in Newman, but not NWM-TS08, during treatment with SXT. Exogenous thymidine suppressed SOS induction by SXT in Newman, signifying a close relationship between SXT lethality and the SOS response. These data indicate that SOS induction plays a role in SXT lethality and suggest that mitigation of this response could contribute to cell survival in the wild type and the *ptsI* mutant.

### A central role for ATP production in lethality and tolerance of SXT in *S. aureus*

ATP is an important cofactor for RecA and the SOS response (56, 57). Several SXT-selected mutations we identified by sequencing, and results from anaerobic SXT challenge (Fig. 4A), suggested that ATP generation may be a critical component of TLD. ATP plays important roles in stress responses and their consequences, including the formation of multidrug-resistant dormant cells known as persisters (59–61). Therefore, we investigated the role of ATP in TLD and whether diminished ATP levels might confer survival of SXT-induced TLD in *S. aureus*. We took advantage of the temporal lag in TLD onset—evident in the growth curves in Fig. 1A and B, where no decrease in cell density was apparent until after 2 h of exposure—to compare the relative dynamics of intracellular ATP and cell density. Using a luciferase-based assay, we measured ATP in wild-type Newman, NWM-TS08, NWM-TS08 + pCN24*-ptsI*, and NWM-TS08 + pCN24 over 2 h of SXT exposure. In the absence of SXT treatment, amounts of ATP were similar between all strains with no significant differences observed during the experiment (Fig. 5B). SXT treatment increased ATP levels in strains with a wild-type *ptsI* gene (Newman and NWM-TS08 + pCN24*-ptsI*) 10.8- and 5.4-fold higher, respectively, compared to the same strains without treatment. ATP levels were significantly higher in SXT-treated, *ptsI* wild-type containing strains (Newman and NWM-TS08-pCN24-*ptsI*) compared to *ptsI* mutant NWM-TS08 without and with empty vector (Fig. 5B). Based on these results, we hypothesized that limiting ATP levels would improve survival against SXT.

To test this hypothesis, we first determined the effect of impairing ATP production on SXT survival in Newman by disrupting electron transport with the proton gradient-uncoupling agent carbonyl cyanide m-chlorophenyl hydrazone (CCCP). The addition of 10 µm CCCP simultaneously halted growth and mitigated SXT-induced cell death compared to no CCCP conditions (Fig. 5C). To more directly test the effects of limiting ATP levels on SXT survival, we repeated the experiment using arsenate, which irreversibly inactivates ATP (62). The effects of 1 mM arsenate on both cell density (Fig. 5C) and ATP production (Fig. S8) in the presence and absence of SXT resembled those of CCCP, providing further evidence that limiting ATP production improves *S. aureus* survival with SXT treatment.

## DISCUSSION

We have shown that *S. aureus* employs several mechanisms to survive the first-line antibiotic combination SXT. Thymidine concentration was not only a major determinant of *S. aureus* viability when treated with SXT, but this nucleoside also influenced the types of mutations that were selected by extended SXT exposure, with either folate metabolic or core metabolic mechanisms predominating depending on thymidine availability. Under thymidine-deplete conditions, survival was influenced by ATP and metabolic rates but not by ROS, in contrast with findings from studies in *E. coli*. These data emphasize not only that *S. aureus* metabolic pathways are malleable but that this type of tolerance, or the emergence of resistance, is highly dependent on the environment.

When investigating the role of thymidine in survival, a minimum concentration of 0.25 µg/mL extracellular thymidine was required for *S. aureus* to survive *in vitro* under clinically relevant SXT concentrations. In Newman, this concentration of thymidine supported the emergence of mutants in the folate metabolic gene *dfrA*, but even higher thymidine concentrations (≥1 µg/mL) were required before mutants in another key folate gene, *thyA,* were detected. *thyA* mutants were not detected in JE2, an observation made previously but remains unexplained (40), suggesting physiological differences between strains. The observation that most CF sputum samples contained thymidine levels <0.25 µg/mL suggests that uptake of exogenous thymidine is not the sole mechanism by which *S. aureus* survives SXT *in vivo*. Gene mutations commonly identified with SXT selection under low-thymidine conditions occurred not in folate metabolism—the target of SXT—but in core metabolic pathways, including glycolysis, the TCA cycle, and electron transport, all of which contribute to ATP production. The selection of electron transport mutants (hemin- and menadione-dependent SCVs) by SXT was a surprising result, as these mutants typically confer aminoglycoside resistance; their ability to promote survival against SXT may explain why ETC mutants are also observed among CF patients treated with that drug (31, 63). Several research groups have described relationships between ATP levels and antibiotic tolerance across diverse bacterial species. For example, in *Pseudomonas aeruginosa*, the disruption of tolerance-promoting genes resulted in increased cellular ATP and decreased persistence (64). In *E. coli*, growth arrest through nutrient starvation or certain bacteriostatic antimicrobials (including trimethoprim) reduced ATP levels and conferred protection during subsequent antibiotic exposure (65). Further work is required to assess the precise roles of glycolysis, TCA cycle, and ETC in *S. aureus* survival to SXT, and if other pathways, such as amino acid catabolism or nucleotide salvage, influence SXT susceptibility or response.

DNA damage and the SOS response have been reported to play important roles in TLD (25, 66), and thymidine starvation likely increases lethality by impairing DNA repair (55, 67). Evidence supporting a role for DNA repair in TLD includes the observation that related enzymes, such as *dnaA* or *mutM* and *mutY* in *E. coli* (20) and *rexAB* and *xerC* in *S. aureus* (25, 66)*,* were required for survival against antifolates. While we did not explicitly quantify DNA damage in this study, our observation of a close relationship between SOS induction, presumably induced by DNA breaks, and SXT lethality suggests a role for DNA damage in SXT action, at least in *S. aureus*. Given the demonstrated importance of ATP for both RecA function and overall SOS responses in other species (56, 57) and the parallels we observed in *S. aureus* survival (observed decreases in SOS induction and ATP in NWM-TS08 with SXT, relative to SXT-treated wild type), ATP may play a relatively complex role in regulating the survival of TLD in a mechanism coupled to DNA repair. Whether SOS induction stimulates higher ATP production or if excess ATP is a prerequisite for SOS induction remains unknown. Further study is required to define the mechanistic details of these events.

It has been proposed that ROS are directly responsible for cell death in an *E. coli* model of TLD (21, 22, 53), although other work with that species contradicts that view (23, 24, 68). Our results indicated no such role for ROS in *S. aureus*. In agreement with the observations by Clarke et al. (25), we observed elevated intracellular ROS with SXT exposure in *S. aureus*. However, we found that chemically inactivating ROS did not improve survival in *S. aureus*, suggesting that, in general, cell death does not require changes in ROS during antibiotic treatment. Taken together, these studies suggest that the nature of ROS involvement in TLD and TLD-like cell death may be more complex and species specific.

**Fig 6:**
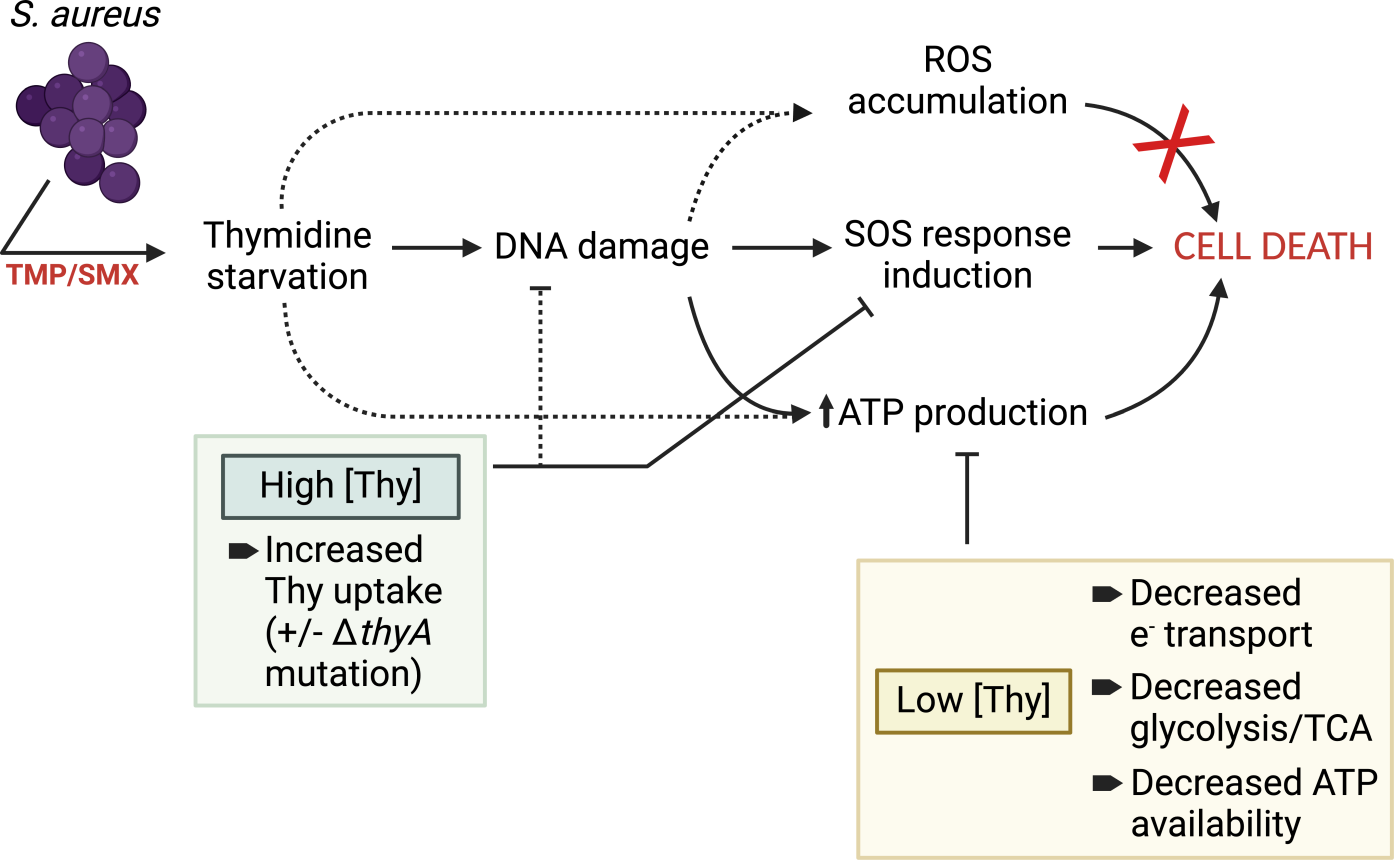
Model of the proposed mechanism of SXT action in *S. aureus*. Upon SXT exposure, *S. aureus* undergoes thymidine starvation and DNA damage, inducing the SOS response and, ultimately, cell death. SOS induction can be mitigated, and survival conferred, by environmental thymidine levels above a threshold of 0.25 µg/mL. In contrast, with continued thymidine depletion, both ROS and ATP levels increase through undefined mechanisms. Of these, only ATP is strongly associated with lethality. Multiple conditions that decrease ATP levels, including decreased electron transport, decreased substrate phosphorylation, or decreased ATP production (either through slow growth or by impairing metabolism or ATP production) improve survival with SXT. Several details of this model remain unknown (dotted lines), including the points at which ROS accumulation and increased ATP production occur, whether thymidine supplementation prevents DNA damage itself, and the mechanism(s) through which ATP levels correlate with lethality. Created in BioRender (L. Gonsalves, 2024, BioRender.com/f49f324)*.*

The results of this study suggest a model of *S. aureus* survival during SXT exposure (Fig. 6) in which thymidine starvation causes DNA damage that induces stress responses, ATP production (57), and ROS generation (perhaps as by-products of increased aerobic respiration) either through independent or interconnected pathways. While elevated ROS abundance is not a major contributor to lethality in *S. aureus*, we propose that increased ATP availability mediates cell death that can be prevented through exogenous thymidine uptake and utilization, reduced SOS response, or mutations or treatments that promote decreased electron transport, core metabolism, and/or ATP availability. In support of this model, we found that manipulating the availability of thymidine or ATP, or dampening growth, each conferred survival of *S. aureus* to SXT in predictable patterns, indicating that even reversible alterations in respiratory activity limit killing by that antibiotic. This work supports a broader, context-dependent paradigm for the mechanism of TLD in bacteria in which environmental conditions, duration of stress exposure, and even the species determine the dominant pathways of bacterial death. These findings therefore indicate the importance of considering the diversity of host tissue conditions (such as nutrient and thymidine concentrations) in designing improved, broad-spectrum antimicrobials targeting pathways related to TLD.

This study had several limitations. While we studied several strains/isolates of *S. aureus,* our experiments were largely conducted *in vitro*, and clinical relevance was not always demonstrated. Specifically, we used artificial media to define thymidine requirements for growth, and the conditions we used for mutant selection may not be uniformly physiologically relevant (for example, our monoculture experimental designs do not accurately model the polymicrobial nature of CF respiratory infections [8, 69]). In addition, our study focused on bacterial determinants of SXT lethality and *S. aureus* survival, while other effects may predominate *in vivo*, such as drug penetration in infected tissues or other nutrients/pressures not accounted for in our model system, potentially limiting generalizability to other infections. Our analysis of thymidine availability *in vivo* was limited to expectorated sputum, a complex mixture of mucus, bacterial cells, bacterial byproducts, and other compounds, such as inflammatory cells, carbon sources, ions, and amino acids (70), which may have variable thymidine concentrations at different locations in the lung. Finally, the *in vivo* relevance of the *in vitro* selected mutations described in this work was not examined and requires a longitudinal analysis of *S. aureus* in SXT-treated individuals relative to a matched, untreated control group. However, the work presented does highlight the complex, non-linear, multi-target nature of SXT mechanism of action and *S. aureus* adaptation, emphasizing how auxiliary pathways, or those not directly targeted, can affect survival. These results expand our understanding of the mechanisms behind SXT tolerance, providing an essential step toward developing improved therapies.

## MATERIALS AND METHODS

### Bacterial strains, growth conditions, reagents, and antibiotics

The strains used in this study are listed in Table S10. *S. aureus* was cultured with Lennox LB agar or broth (BD Difco) at 35°C. Mass spectrometry analysis (as described in the methods below) of two different lots of LB found thymidine amounts were at or below the level of quantitation (0.041 µg/mL) and insufficient to support *thyA* growth or antagonize SXT activity without supplementation. ∆*thyA* was cultured on LB agar supplemented with a final concentration of 1.5 µg/mL thymidine. Liquid cultures were grown with agitation (225 rpm). Where indicated, media were supplemented with a final concentration of 0–16 µg/mL thymidine, 10 µM CCCP, or 1 mM sodium arsenate dibasic heptahydrate (all Sigma-Aldrich) for survival assays. Antibiotics used in survival assays and cloning included trimethoprim-sulfamethoxazole at 8/152 µg/mL, 10 µg/mL erythromycin (transposon maintenance), 50 µg/mL ampicillin, or 50 µg/mL kanamycin. For enumeration, blood and chocolate agar plates were used (ThermoFisher).

### SXT survival assays

Overnight cultures of *S. aureus* were diluted in fresh LB broth to a final density of 1 × 10^6^ CFU/mL. Cultures were incubated with SXT and supplements as indicated. Cultures were serially diluted in 1× PBS (pH 7.4; Gibco) and plated for viable counts at specific time points. For anaerobic experiments, initial starting cultures and all reagents and media were deoxygenated overnight where possible, and all work was conducted in a Coy anaerobic chamber.

### Enrichment of adaptive mutants

Experiments to select for adaptive mutants were performed as survival assays except that cultures were backdiluted in LB with fresh thymidine supplementation and SXT addition every 24 h, at which point aliquots of culture were removed for DNA extraction. Representative colonies of diverse morphologies on LB, chocolate, and blood agar were selected for phenotyping and sequencing.

### DNA extraction, whole-genome and population-level sequencing, and analysis

DNA was extracted from cultures using the Qiagen DNeasy UltraClean Microbial Kit (Qiagen) as recommended by the manufacturer except that lysostaphin was added to the resuspended pellet at a final concentration of 0.14 mg/mL and incubated at 37°C for 30 min; the volume of solution SL added was then adjusted to account for the additional volume, followed by light vortexing and incubation at 70°C for 10 min.

Sequencing libraries were prepared as previously described (71), followed by sequencing on the NextSeq500 (Illumina) with 300-cycle chemistries. The sequencing depth for isolates was at least 43× coverage. Reads were analyzed as previously described (72), except using BWA-MEM (version 0.7.12) (73) to align sequences to appropriate reference genomes (Newman, GenBank accession AP009351.1; JE2, GenBank accession CP000255.1). SAMtools (version 1.1) was used for variant calling (74). Mutations were annotated with sequence features using SnpEff (75). Average sequencing depth for population sequencing was 8.1 million reads per specimen (ranges from 2.1 to 41.2 million reads), which were mapped as described above, followed by SNP variant calling using LoFreq2 “call” algorithm (76) and SnpEff for mutation annotation. Upstream and synonymous variants were filtered out, and the additive variant allele frequency for each gene was calculated (aVAF; the total proportion of unique, nonsynonymous mutations). Genes with mutations detected in control experiments were excluded from further analysis, and the average aVAF for each experimental condition by time point (high thymidine, 1, 4, and 1 µg/mL; low thymidine, 0, 0.06, and 0.25 µg/mL) across available replicates was determined.

### Auxotrophic assays

Colony phenotypes and auxotrophy were determined as previously described (30).

### Liquid chromatography-tandem mass spectrometry of sputum samples

Fifty microliters of sputolysin-treated (Calbiochem) CF sputum samples was analyzed for the amounts of thymidine and its analogs by mass spectrometry using standard curves generated for thymidine (Fisher) and deoxythymidine monophosphate (dTMP; Sigma), diphosphate (dTDP; Fisher), or triphosphate (dTTP; Sigma). Ten microliters of C13 MTP internal standard (Sigma; 100 ng/mL) was added followed by 150 µL of acetonitrile. Samples were centrifuged at 16.1 rcf for 5 min. Samples were reconstituted with 50 µL of mobile phase/solvent A, 0.1 M ammonia acetate in water, pH 9.5 (Sigma Aldrich). Twenty microliter sample injections were analyzed on a Water’s Xevo TQ-s triple quadrupole mass spectrometer, coupled to a Water’s Acquity I-Class Ultra high-pressure liquid chromatography system in negative ionization mode (Neg-ESI) with the following transitions for each analyte: thymidine, 241.2 to 125.1 and 151.2; dTMP, 321.2 to 125.2 and 195.2; dTDP, 401.2 to 159.0 and 275.2; and dTTP, 481.0 to 159.0 and 383.1. IS C13 MTP exhibited a transition of 333.2 to 182.0 and 200.0. Separation was achieved using a Thermo Hypercarb 2.1 × 50 mm, 3µ column with a 0.2um frit as a guard and a flow rate of 0.3 mL/min with gradients listed in Table S11.

### Strain construction

*ptsI* complementation of mutant NWM-TS08 and *RecA*-GFP reporter strains were constructed essentially as described (77). For the *ptsI* complementation mutant, the target gene, preceded by a *sarA* promoter and *sod* RBS (78), was constructed by spliced overlap extension PCR and the primers SbfI-*sarA* promoter forward, *sarA* promoter-*sod* RBS internal reverse, *sod* RBS-*ptsI* internal forward, and *ptsI*-KpnI reverse (Table S10). For the construction of the RecA reporter strain, a gBlock *recA*-GFP insert with restriction enzyme sites was designed as described (79, 80) and constructed by IDT. The insert fragments were cloned into restriction sites *KpnI* and *SbfI* of pCN34 (BEI resources, strain NR-46121), transformed into NEB 10-beta competent *E. coli* cells, followed by RN4220, and finally transformed into *S. aureus* strain Newman and mutant isolate NWM-TS08 by electroporation.

### Measurement of intracellular ROS and ROS mitigation assays

CM-H_2_DCFDA (ThermoFisher, excitation ~492–495 nm and emission 517–527 nm) was reconstituted to 1 mM in DMSO and used to quantify ROS in cultures as recommended by the manufacturer with modifications. Briefly, reconstituted dye was added to 1 × 10^7^ CFU/mL-containing culture at 5 µM alongside other supplements and SXT and incubated for 20 min. Following initial incubation, 1–2 mL of culture was removed to establish an intracellular ROS baseline, and the remaining cultures were incubated. At specific time points, 1–3 mL aliquots were removed from the culture, centrifuged at 6,500 × *g* for 5 min, pellets were washed with 1× PBS, and resuspended in 1× PBS. Samples were analyzed on a BD Accuri C6 Plus with a flow speed of 14 µL/min and a core size of 10 µm was used. Fluorescence was detected using a 533/30 standard optical filter (FL1), and data were analyzed using FlowJo software (version 10.8.1). ROS mitigation assays were prepared similarly to survival assays except with the addition of Trolox (Cayman Chemical) to cultures at a final concentration of 0.2 mM. Intracellular ROS was analyzed via BioTek Synergy H1 Hybrid reader.

### Measurement of relative ATP

ATP was quantified in cultures using the BacTiter-Glo Microbial Cell Viability Kit (Promega), according to the manufacturer’s instructions.

### SOS induction assay

Overnight cultures of strain Newman harboring the pCN34-RecA-GFP plasmid were diluted to ~1 × 10^6^ CFU/mL in LB with kanamycin for plasmid maintenance and treated with either 0.005 µg/mL mitomycin C, 128 µg/mL of thymidine, and/or varying concentrations of subinhibitory levels of SXT (0.125–0.5 µg/mL), and incubated at 35°C with constant agitation. Fluorescent data and OD_600_ were measured with a BioTek Synergy H1 Hybrid reader.

## Data Availability

Data analysis and figure construction were performed with GraphPad Prism 9, version 10.1.1. Sequencing data from this study are publicly available through NCBI Sequence Read Archive under the BioProject ID PRJNA1083920.

## SUPPLEMENTAL MATERIAL

Supplemental Figures mbio.01634-24-s0001.docx Figures S1-S4.

Supplemental Figures mbio.01634-24-s0002.docx Figures S5-S8.

Supplemental Tables mbio.01634-24-s0003.xlsx Tables S1-S11.

ASM does not own the copyrights to Supplemental Material that may be linked to, or accessed through, an article. The authors have granted ASM a non-exclusive, world-wide license to publish the Supplemental Material files. Please contact the corresponding author directly for reuse.
