# Supplementary material for: Mechanisms of Staphylococcus aureus survival of trimethoprim-sulfamethoxazole-induced thymineless death
Source: mBio. 2024 Oct 24;15(11):e01634-24. doi: 10.1128/mbio.01634-24 (PMC11559000; doi:10.1128/mbio.01634-24)
Supplement: Supplemental Figures — Figures S1-S4. [file mbio.01634-24-s0001.docx]

**Mechanisms of *Staphylococcus aureus* survival of trimethoprim-sulfamethoxazole-induced thymineless death**

Lauren J. Gonsalves, Allyson Tran, Tessa Gardiner, Tiia Freeman, Angshita Dutta, Carson J. Miller, Sharon McNamara, Adam Waalkes, Dustin R. Long, Stephen J. Salipante, Lucas R. Hoffman, and Daniel J. Wolter*

**Supplemental Figures S1-S4**

Figure S1. Kinetics of survival of *S. aureus* strain Newman in the presence and absence both of trimethoprim-sulfamethoxazole (SXT) and delayed thymidine supplementation.

Figure S2. Colony phenotypic diversity of *S. aureus*isolates selected by short-term (24h) SXT exposure.

Figure S3. Survival of ∆*thyA* and wild-type Newman undergoing TLD in the presence of thymidine and analogs.

Figure S4. Survival kinetics of clean deletion strains or transposon mutants to SXT challenge.


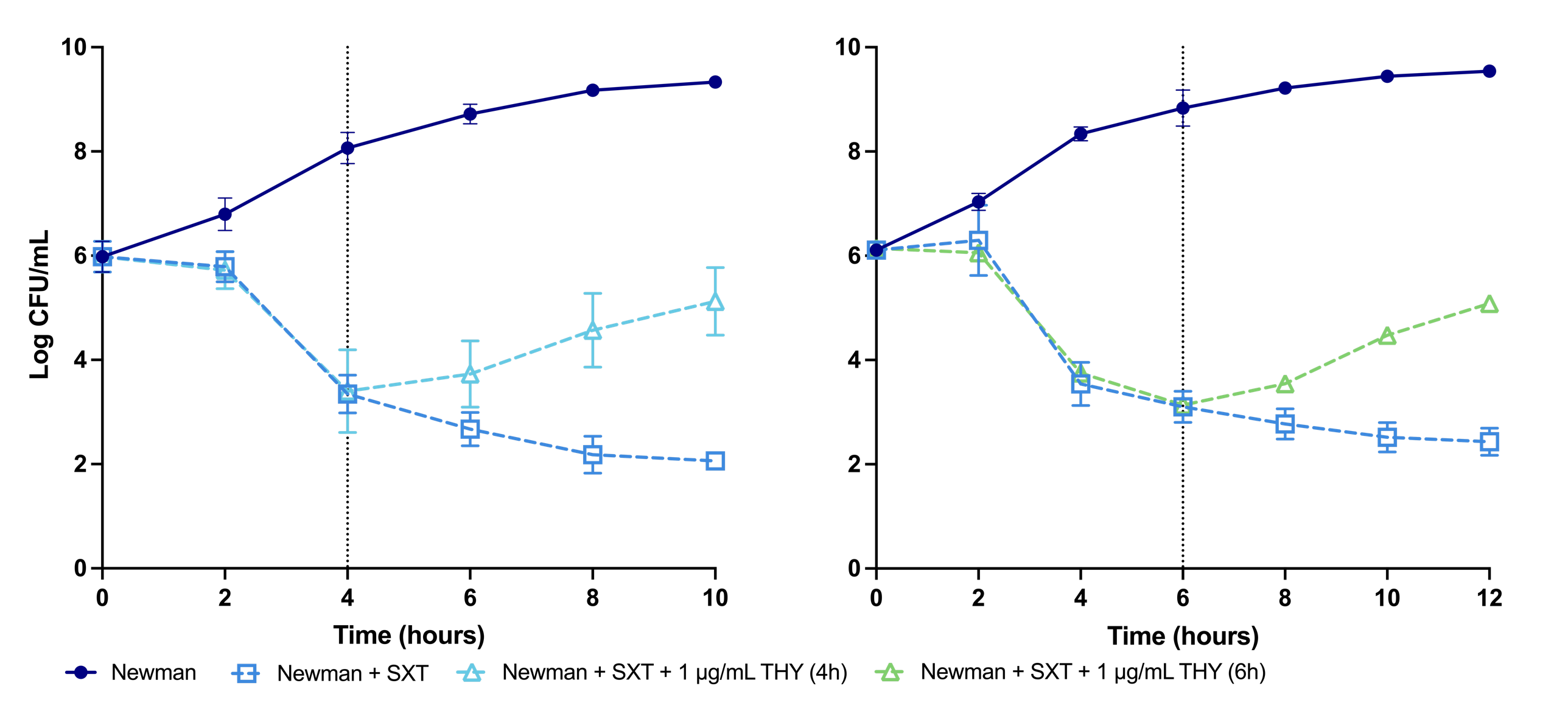


**Figure S1.** **Kinetics of survival of *S. aureus* strain Newman in the presence and absence both of trimethoprim-sulfamethoxazole (SXT) and delayed thymidine supplementation.** *S. aureus* strain Newman was cultured in LB over 10-12h. The culture was supplemented with 1 µg/mL thymidine added at either 4h (dotted vertical line, left) or 6h (dotted vertical line, right) after initiation of SXT treatment. SXT-treated conditions are indicated by a dotted horizontal line; data are mean ± SD (n=3) and treated with 8 µg/mL TMP and 152 µg/mL SMX.

**
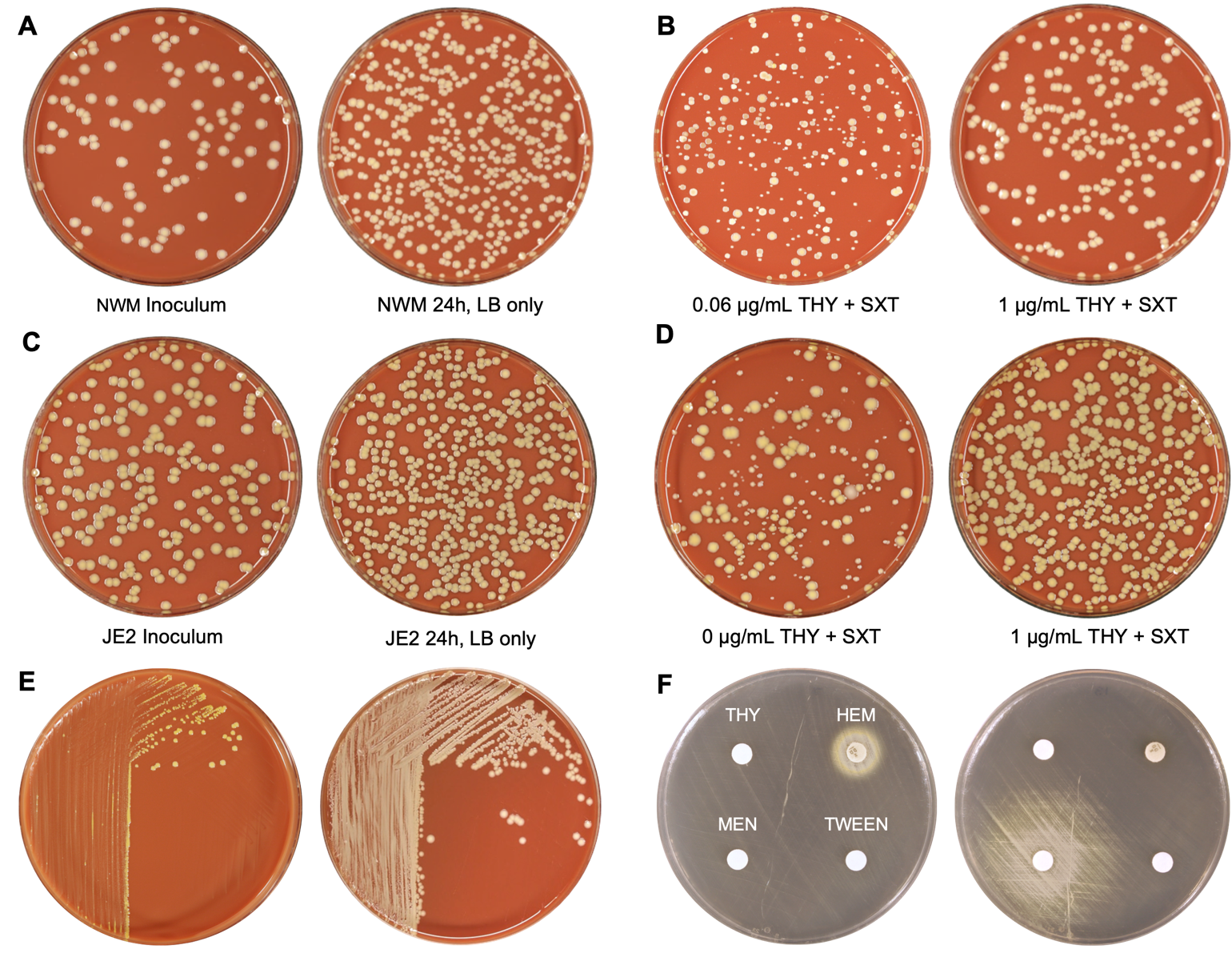
**

**Figure S2. Colony phenotypic diversity of *S. aureus*isolates selected by short-term (24h) SXT exposure. (A)** Colony morphologies of plated Newman inoculum before (left) and after 24h growth (right) in LB only, revealing homogeneous morphology. **(B)** Representative plates following 24h of SXT treatment of Newman from thymidine conditions 0.06 µg/mL (left) and 1 µg/mL (right) exhibiting diverse colony morphology and normal colony phenotype, respectively. **(C)** Result as in (A) but with JE2 inoculum. **(D)** Representative plates following 24h of SXT treatment of JE2 from thymidine conditions 0 µg/mL (left) and 1 µg/mL (right) exhibiting diverse colony morphology and normal colony phenotype, respectively. **(E)** Plate of representative Newman colonies exhibiting hyperpigmentation (increased staphyloxanthin production; left) following SXT exposure; plate of representative normal colonies for reference (right). **(F)** Representative auxotrophic testing results of select Newman isolates following 24h of treatment with SXT under low thymidine conditions. Plates show growth around discs saturated with hemin and menadione (respectively), indicating auxotrophies complemented with supplementation.


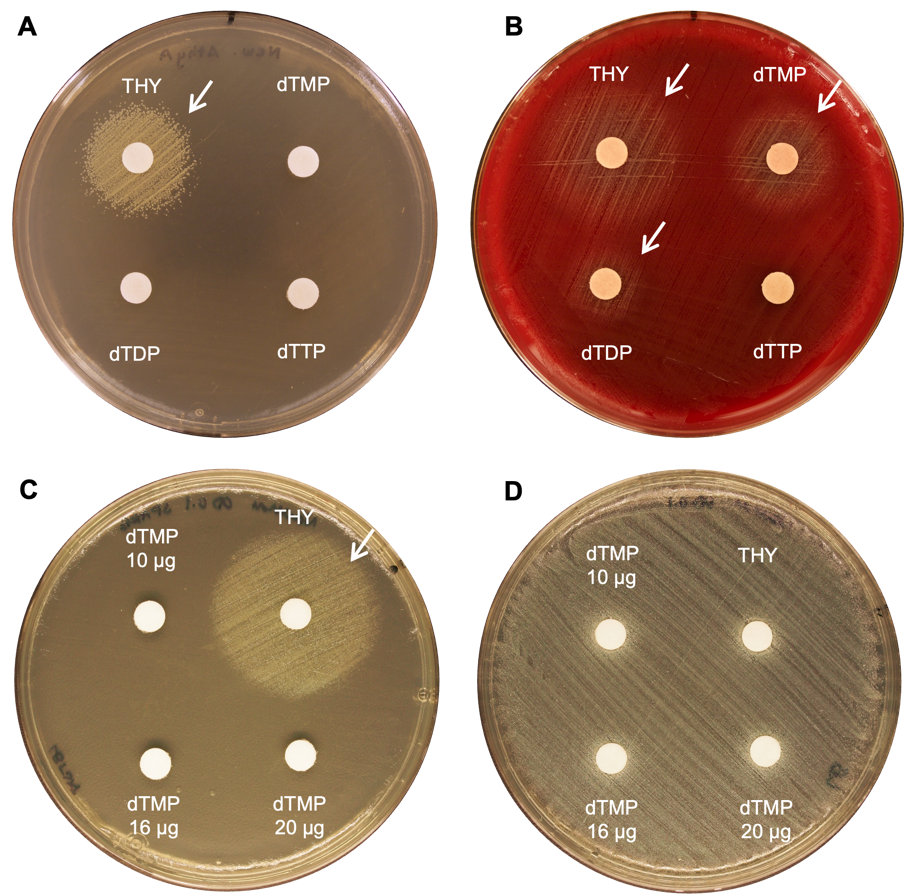


**Figure S3. Survival of ∆*thyA* and wild-type Newman undergoing TLD in the presence of thymidine and analogs.** Auxotrophic testing with thymidine (THY) and analogs deoxythymidine-monophosphate, -diphosphate, and -triphosphate (dTMP, dTDP, and dTTP, respectively) for Newman ∆*thyA* grown on **(A)** LB and **(B)** blood agar plates; all disks were saturated with 10 µg of the respective compound. Representative auxotrophic testing of thymidine (10 µg) and dTMP (10-20 µg) for *S. aureus* wild-type strain Newman on **(C)** LB + SXT and **(D)** LB.





**Figure S4. Survival kinetics of clean deletion strains or transposon mutants to SXT challenge.** Survival kinetics of **(A)** Newman ∆*menB* and **(B)** Newman ∆*hemB*, cultured in LB over 10h in the absence of thymidine with and without SXT and with or without menadione or hemin (1 µg/mL), where indicated. Data are mean ± SD (n≥2). **(C)** Survival kinetics of JE2 *hepT*::Tn cultured in LB over 10h in the absence of additional thymidine and treated with SXT. SXT-treated conditions are indicated by a dotted line and data are mean ± SD (n=3).
