# Supplementary material for: Mechanisms of Staphylococcus aureus survival of trimethoprim-sulfamethoxazole-induced thymineless death
Source: mBio. 2024 Oct 24;15(11):e01634-24. doi: 10.1128/mbio.01634-24 (PMC11559000; doi:10.1128/mbio.01634-24)
Supplement: Supplemental Figures — Figures S5-S8. [file mbio.01634-24-s0002.docx]

**Mechanisms of *Staphylococcus aureus* survival of trimethoprim-sulfamethoxazole-induced thymineless death**

Lauren J. Gonsalves, Allyson Tran, Tessa Gardiner, Tiia Freeman, Angshita Dutta, Carson J. Miller, Sharon McNamara, Adam Waalkes, Dustin R. Long, Stephen J. Salipante, Lucas R. Hoffman, and Daniel J. Wolter*

**Supplemental Figures S5-S8**

Figure S5. Survival kinetics of *S. aureus* during SXT treatment in dilute media.

Figure S6. Survival of the *S. aureus* *ptsI* mutant complemented with wild-type *ptsI* during SXT challenge.

Figure S7. Total reactive oxygen species levels present in *S. aureus* in experiments supplemented with the ROS scavenging compound Trolox.

Figure S8. Whole-culture ATP levels with SXT treatment and either arsenate (ars) or CCCP.


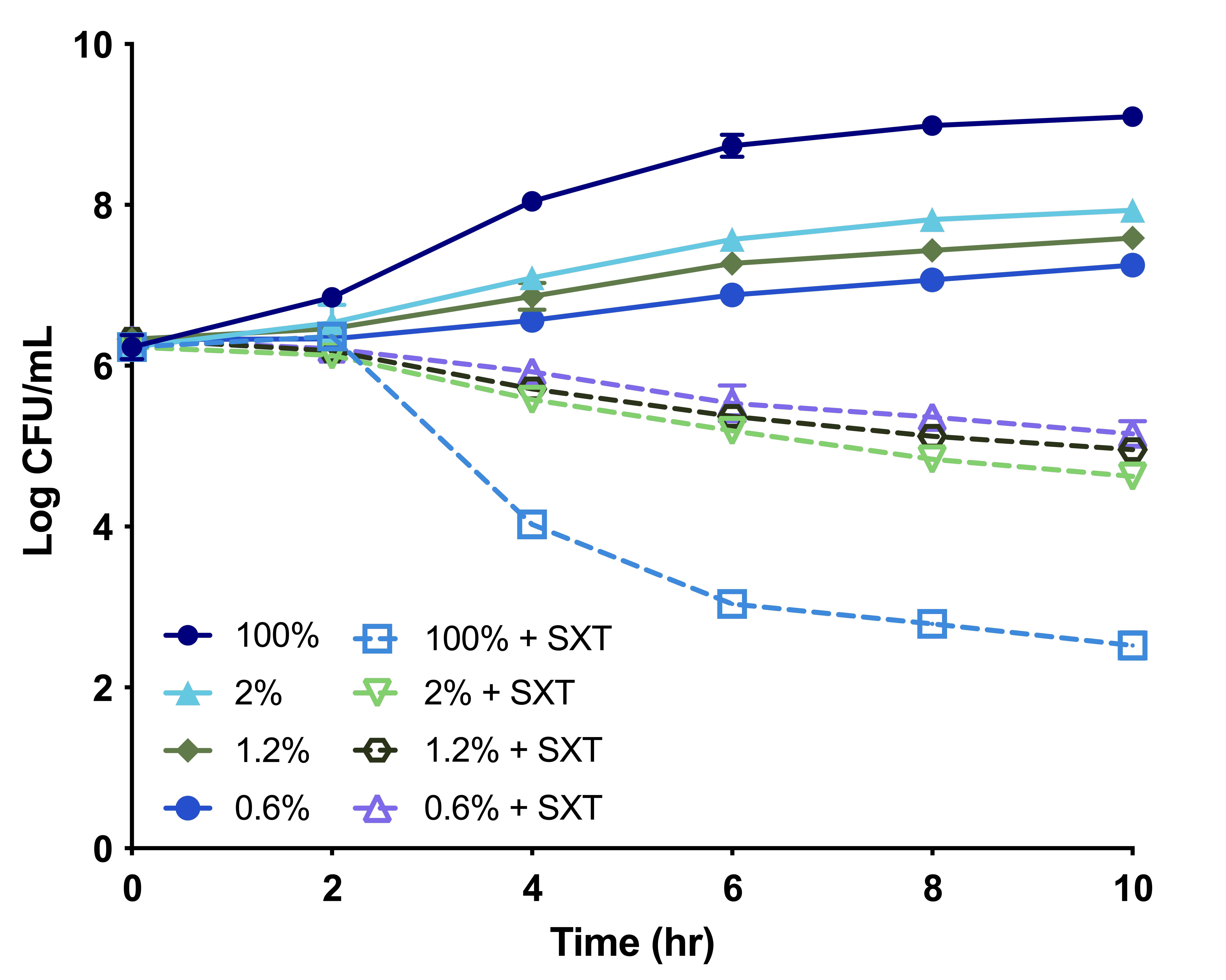


**Figure S5. Survival kinetics of *S. aureus* during SXT treatment in dilute media.** *S. aureus* Newman was cultured in 100% or dilute LB (2%, 1.2% and 0.6% LB, as indicated) containing 50 mM MOPS (pH 7.0) with and without SXT over 10h; SXT-treated conditions are indicated by a dotted line and shown are mean ± SD (n=3).


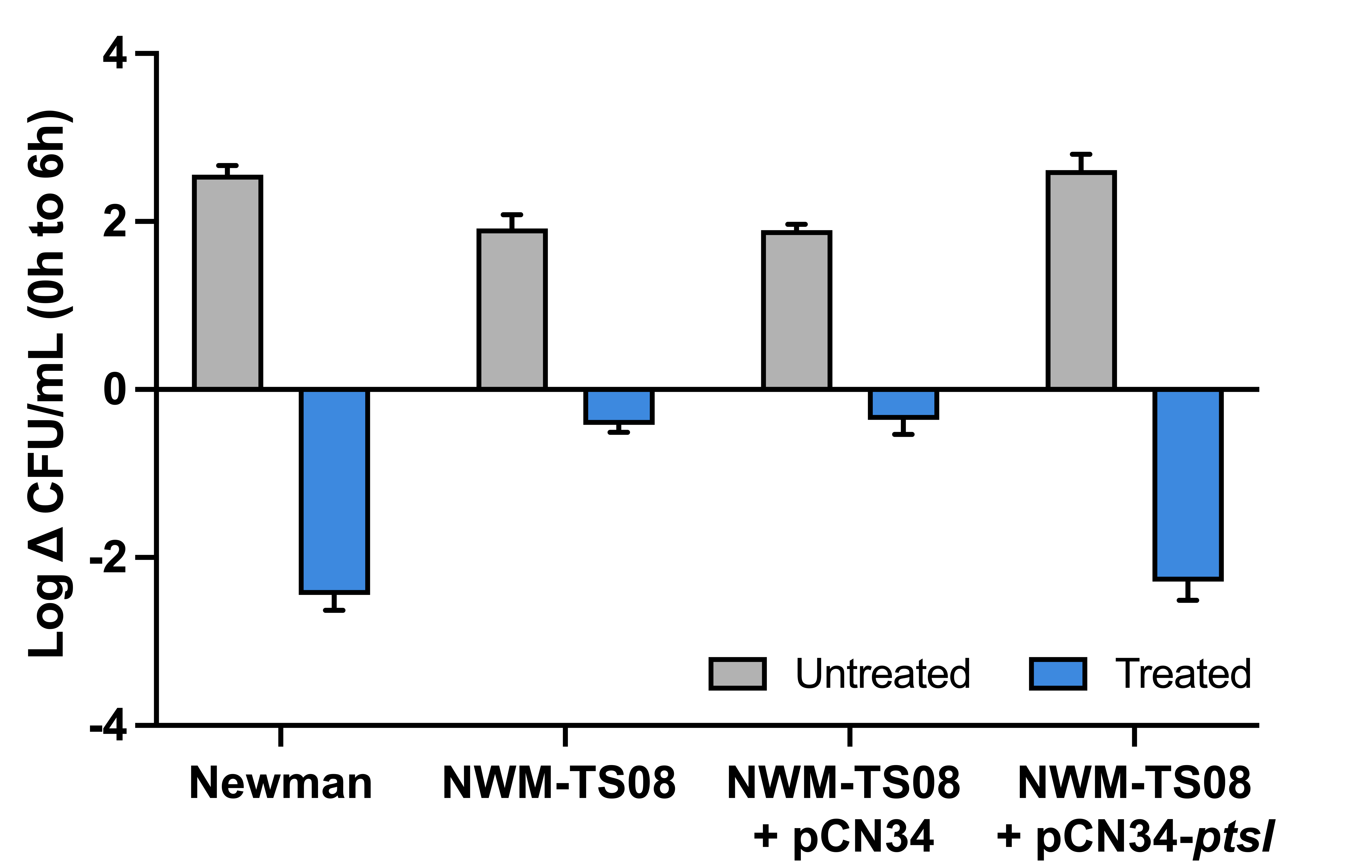


**Figure S6. Survival of the *S. aureus* *ptsI* mutant complemented with wild-type *ptsI* during SXT challenge.** Values on the y-axis are expressed as change in Log CFU/mL for parental strain Newman, *ptsI* mutant NWM-TS08, and NWM-TS08 complemented with wild-type *ptsI* after 6h with or without SXT treatment relative to 0h timepoint. Data represent mean ± SD (n=3).


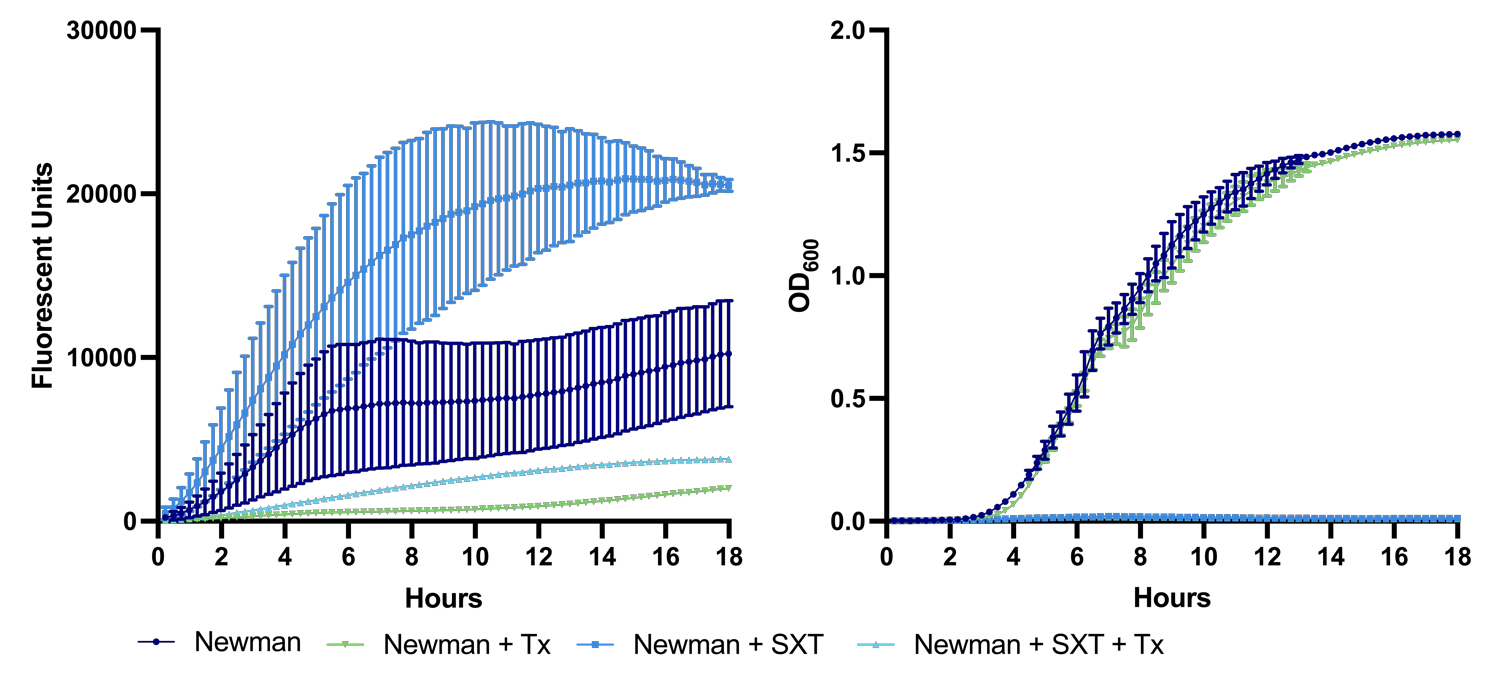


**Figure S7. Total reactive oxygen species levels present in *S. aureus* in experiments supplemented with the ROS scavenging compound Trolox.** S*. aureus* Newman treated with SXT, CMH_2_DCFDA, and Trolox and analyzed via spectrophotometer for ROS levels over 18h. Data presented as fluorescence (left) and OD_600_ (right) over time; data are mean ± SD (n=2).


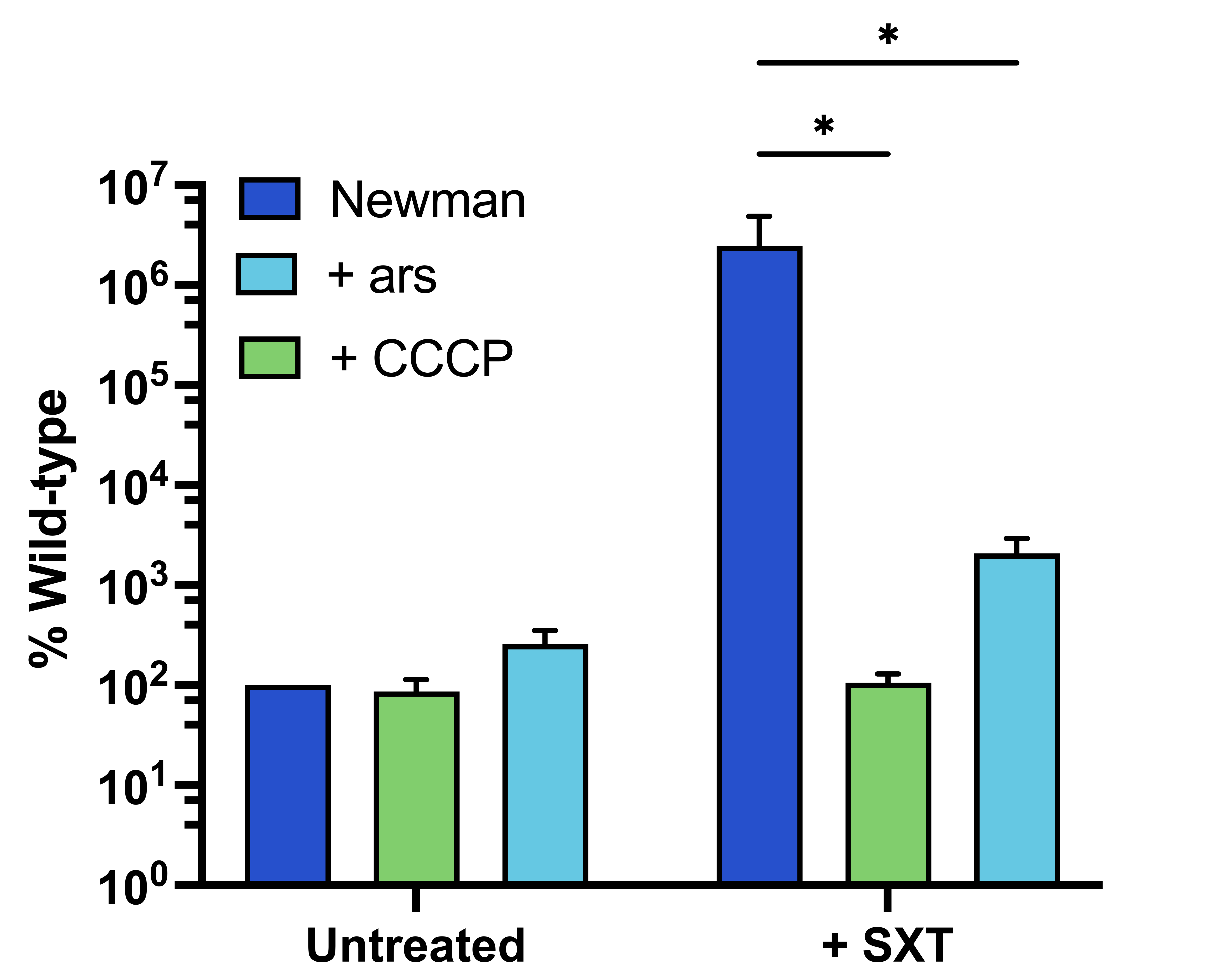


**Figure S8. Whole-culture ATP levels with SXT treatment and either arsenate (ars) or CCCP.** Relative ATP levels at 6h post-SXT exposure (RLU normalized to CFU/mL) presented as percent of wild-type for treated and untreated Newman and *ptsI* mutant NWM-TS08. The ratio of treated to untreated condition for Newman only, Newman with arsenate (ars), and Newman with CCCP is 24,765.47, 1.23, and 8.03, respectively; data are mean ± SD (n=3) and significance (*) reported where p-value ≤ 0.05, as determined via a two-way ANOVA.
